# Occurrence and Impact of Gastrointestinal Bleeding and Major Adverse Cardiovascular Events during Sepsis: A 15-Year Observational Study

**DOI:** 10.1155/2020/9685604

**Published:** 2020-09-27

**Authors:** Ming-Shun Hsieh, Shu-Hui Liao, Vivian Chia-Rong Hsieh, Chorng-Kuang How

**Affiliations:** ^1^Department of Emergency Medicine, Taipei Veterans General Hospital, Taoyuan Branch, Taoyuan, Taiwan; ^2^Department of Emergency Medicine, Taipei Veterans General Hospital, Taipei, Taiwan; ^3^School of Medicine, National Yang-Ming University, Taipei, Taiwan; ^4^Department of Pathology and Laboratory, Taipei Veterans General Hospital, Taoyuan Branch, Taoyuan, Taiwan; ^5^Department of Health Services Administration, China Medical University, Taichung, Taiwan

## Abstract

**Objective:**

Sepsis patients are at risk of gastrointestinal bleeding (GIB) and major adverse cardiovascular events (MACEs), but few data are available on the occurrence of GIB and MACEs and their impact on sepsis outcomes.

**Methods:**

The medical claims records of 220,082 patients admitted for sepsis between 1999 and 2013 were retrieved from the nationwide database. The adjusted odds ratios (aORs) of composite outcomes including the hospital mortality, intensive care unit (ICU) admission, and mechanical ventilation (MV) in patients with a MACE or GIB were estimated by multivariate logistic regression and joint effect analyses.

**Results:**

The enrollees were 70.15 ± 15.17 years of age with a hospital mortality rate of 38.91%. GIB developed in 3.80% of the patients; MACEs included ischemic stroke in 1.54%, intracranial hemorrhage (ICH) in 0.92%, and acute myocardial infarction (AMI) in 1.59%. Both ICH and AMI significantly increased the risk of (1) ICU admission (aOR = 8.02, 95% confidence interval (CI): 6.84–9.42 for ICH and aOR = 4.78, 95% CI: 4.21–5.42 for AMI, respectively), (2) receiving MV (aOR = 3.92, 95% CI: 3.52–4.40 and aOR = 1.99, 95% CI: 1.84–2.16, respectively), and (3) the hospital mortality (aOR = 1.08, 95% CI: 0.98–1.19 and aOR = 1.11, 95% CI: 1.03–1.19, respectively). However, sepsis with GIB or ischemic stroke increased only the risk of ICU admission and MV but not the hospital mortality (aOR = 0.98, 95% CI: 0.93–1.03 for GIB and aOR = 0.84, 95% CI: 0.78–0.91 for ischemic stroke, respectively).

**Conclusions:**

GIB and MACEs significantly increased the risk of ICU admission and receiving MV but not the hospital mortality, which was independently associated with both AMI and ICH. Early prevention can at least reduce the complexity of clinical course and even the hospital mortality.

## 1. Introduction

Sepsis is a complex syndrome induced by severe infection and involving acute organ failure [1]. Despite advances in drugs and treatment modalities, management of sepsis patients is a critical care challenge [2, 3]. Decreasing the occurrence of sepsis-associated complications would be expected to improve hospital mortality and the clinical course by reducing the need for intensive care unit (ICU) admission and mechanical ventilation (MV) support. Few data on the incidence and impact of major adverse cardiovascular events (MACE) and gastrointestinal bleeding (GIB) in sepsis patients are available even though the concurrent development of MACE and GIB in sepsis patients is not unusual [4].

An analysis of over 119,000 patients hospitalized with sepsis between 2003 and 2012 and included in a nationwide database in the USA estimated that the incidence of GIB was 5.4% (6,571/119,684 patients). Concurrent GIB was found to increase sepsis mortality by 9% [5]. Sepsis-associated atrial fibrillation, coagulopathy, hemodynamic instability, and prolonged systemic inflammation act to trigger acute ischemic stroke. Ischemic stroke events are not unusual in patients with sepsis and thrombocytopenia, but the cause appears to be complex [6–9].

An analysis of data from over 2.6 million sepsis patients included in a national inpatient database in the USA from 2002 to 2011, found that 4.5% (118,183/2,602,854 patients) had a concurrent, nonprimary diagnosis of AMI during hospitalization. Non-ST-elevation AMI was the diagnosis of 71.4% of those cases. Hospital mortality was higher in sepsis patients with AMI (35.8%) than those with sepsis alone (16.8%, *P* < 0.001; adjusted odds ratio (aOR) = 1.24, 95% confidence interval (95% CI): 1.22–1.26). Invasive management concurrent AMI was associated with reduced mortality compared with conservative management (OR 0.47, 95% CI: 0.44–0.50) [10].

The treatment of sepsis patients with concurrent GIB and a MACE is complicated by difficulties in choosing among antiplatelet, anticoagulation, and hemostasis drugs. The use of predisposing medications such as antiplatelet drugs, anticoagulants, and proton pump inhibitors (PPIs) before or during the course of sepsis also complicates treatment.

This study used the 15-year nationwide database of Taiwan that included data from 1999 to 2013. The data were from 220,082 patients who were first admitted for sepsis to determine the frequency of occurrence of GIB and MACEs in the course of sepsis. The impacts and interactions of MACE and GIB on the composite outcomes of the hospital mortality, ICU admission, and receiving MV were analyzed.

## 2. Methods

### 2.1. Data Source

The study database included anonymized patient and claims information retrieved from the National Health Insurance Research Database (NHIRD) of Taiwan. The records of 220,082 inpatients who were first admitted with a diagnosis of sepsis between 1999 and 2013 were included in the analysis. The NHIRD is maintained by the National Health Insurance Program, which was launched by the National Health Insurance Administration (NHIA) in 1995, and currently provides coverage for more than 23.03 million residents (>99% of the entire population). The NHIRD included the data from the clinic, district hospital, regional hospital, and medical center. The confidentiality and quality of the NHIRD data have been documented in previous studies [11–14].

### 2.2. Study Participants

Sepsis patients were identified by ICD-9-CM discharge diagnosis code 038 from the NHIRD. The positive predictive value of the sepsis (92.3%) and septic shock (97.0%) diagnoses have been previously validated [12, 15].

All the enrolled sepsis patients should include a main diagnosis coding of sepsis in the first or second diagnostic coding plus a coding representing the infection origin within the first three diagnoses. The infection origin coding was referred to Angus et al. in 2001 [16]. Besides, GIB or MACE could not be the first diagnosis code or have been entered before a diagnosis code for sepsis [17, 18].

### 2.3. MACEs and GIB

The MACEs were defined by referring to International Classification of Disease, Ninth Revision, Clinical Modification (ICD-9-CM), as the compositions of acute myocardial infarction (AMI) (ICD-9-CM 410), ischemic stroke (ICD-CM-9: 433,434.1, 434.9, and 435) and intracranial hemorrhage (ICH) (ICD-9-CM: 430.xx, 431.xx, 767.0, and 772.2). GIB was defined by ICD-CM-9: 578.9.

### 2.4. Potential Confounders

We systematically identified the potential confounders in the claims data. The identified confounding factors were age, sex, insurance premium (as a proxy of household income), level of urbanization, baseline comorbidities, and medications. The baseline comorbidities were (1) hypertension (HTN) (ICD-9-CM: 401–405), (2) diabetes mellitus (DM) (ICD-9-CM: 250, 357.2, 362.01, 362.02, and 366.41), (3) congestive heart failure (CHF) (ICD-9-CM: 402.01, 402.11, 402.91, 404.01, 404.03, 404.11, 404.13, 404.91, 404.93, and 428.0), (4) chronic obstructive pulmonary disease (COPD) (ICD-9-CM: 490, 491, 492, 494, and 496), (5) chronic liver disease (CLD) (ICD-9-CM: 571), (6) chronic kidney disease (CKD) (ICD-9-CM: 581–588, 403–404, 285.21, and 250.4), and (7) cancer (ICD-9-CM: 140–208). Drug use was identified by claims indicating use for more than 1 week within a one-year period prior to the index date.

### 2.5. Selection Process

Patients <18 or >100 years of age or infected with human immunodeficiency virus were excluded from the study. In the patients with repeated admissions, only data from the first hospitalization for sepsis between 1999 and 2013 were included in the analysis. The date of admission for the first hospitalization for sepsis was defined as the index date. Comorbidities were identified by ICD-9-CM codes of diagnoses made within a one-year period prior to the index date.

### 2.6. Ethical Approval

As the database contained deidentified data for research, the study was exempted from obtaining informed consent from the participants. This study was approved by the Institutional Review Board of Taichung Veterans General Hospital (CE18102A) and China Medical University (CMUH104-REC2-115).

### 2.7. Statistical Analysis

Differences in demographic characteristics, baseline comorbidities, drug use (including aspirin, clopidogrel, warfarin, metformin, nonsteroidal anti-inflammatory drugs, statins, PPIs, steroids, and immunosuppressants), and the composite outcomes (total hospital mortality, ICU admission, and MV) were compared by the chi-squared or two-sample *t*-test.

Odds ratios (ORs) with 95% confidence intervals (CIs) were calculated for each variable in the logistic regression model. Adjusted ORs (aORs) for total hospital mortality, ICU admission, and receiving MV were obtained after adjusting for potential confounders including age, sex, insurance premium (a proxy for household income), urbanization level (a proxy for the accessibility of medical care), and comorbidities [15]. The Kaplan–Meier analysis was conducted to compare the cumulative incidence of hospital mortality between the patients with and without GIB and MACE, respectively.

Joint effect analysis was used to analyze the synergistic impact of sepsis complications including GIB, ischemic stroke, ICH, and AMI, on total hospital mortality, ICU admission, and MV. The 16 possible combinations of the four complications were evaluated using uncomplicated sepsis as the reference. The aORs of each combination of complications were calculated by logistic regression after adjusting for age, sex, insurance premium, urbanization level, and baseline comorbidities.

The statistical analysis was performed with SAS 9.4 (SAS Institute, Inc., Cary, NC, USA). *P* values ≤0.05 were considered significant.

## 3. Results

### 3.1. Demographic Characteristics, Baseline Comorbidities, and Clinical Presentation

After exclusion, a total of 220,082 patients with a first admission for sepsis between 1999 and 2013 were retrieved from the nationwide database. The patient characteristics are shown in Table 1. The mean age was 70.15 ± 15.17 years and 56.39% was men. Hypertension was the most common comorbidity (68.31%), followed by diabetes mellitus (DM, 62.19%) and chronic obstructive pulmonary disease (45.64%). The most frequent medications were PPIs (41.73%), aspirin (13.11%), and clopidogrel (8.03%). Septic shock developed in 50.78% of the patients (111,754/220,082), and total hospital mortality was 38.91% (85,638/220,082). The clinical course of sepsis was accompanied by GIB in 3.80%, ischemic stroke in 1.54%, ICH in 0.92%, and AMI in 1.59% of cases. The origins of sepsis were primarily respiratory system (39.87%), genitourinary (30.22%), and gastrointestinal/biliary-tract (8.09%) infections.

### 3.2. Logistic Regression Analysis of Total Hospital Mortality, ICU Admission, MV, and Complications of Sepsis

After adjusting for age, sex, insurance premium, urbanization level, and baseline comorbidities, the aOR of GIB for total hospital mortality was 0.98 (95% CI, 0.93–1.03), the aOR for ICU admission was 1.30 (95% CI, 1.23–1.38), and the aOR for MV was 1.32 (95% CI, 1.26–1.40) with uncomplicated sepsis as the reference (Table 2). Ischemic stroke was associated with an increased risk of ICU admission (aOR = 2.71, 95% CI, 2.47–2.97) and MV (aOR = 2.07, 95% CI, 1.90–2.25) but did not affect the risk of hospital mortality (aOR = 0.84, 95% CI, 0.78–0.91). ICH and AMI had similar effects on sepsis outcomes. In complicated sepsis, ICH increased the risk of total hospital mortality (aOR = 1.08, 95% CI, 0.98–1.19), ICU admission (aOR = 8.02, 95% CI, 6.84–9.42), and MV (aOR = 3.92, 95% CI, 3.52–4.40) compared with uncomplicated sepsis. The corresponding aORs for AMI were 1.11 (95% CI, 1.03–1.19) for total hospital mortality, 4.78 (95% CI, 4.21–5.42) for ICU admission, and 1.99 (95% CI, 1.84–2.16) for MV.

### 3.3. Kaplan–Meier Analysis with the Log-Rank Test

**Figure 1:**
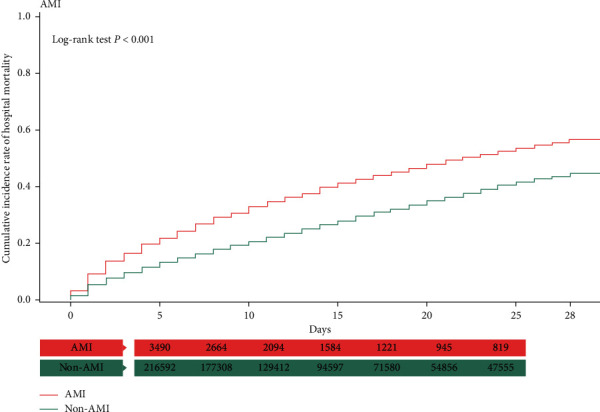
Kaplan–Meier analysis of cumulative hospital mortality in sepsis patients with and without AMI. The differences were evaluated by the log-rank test.

**Figure 2:**
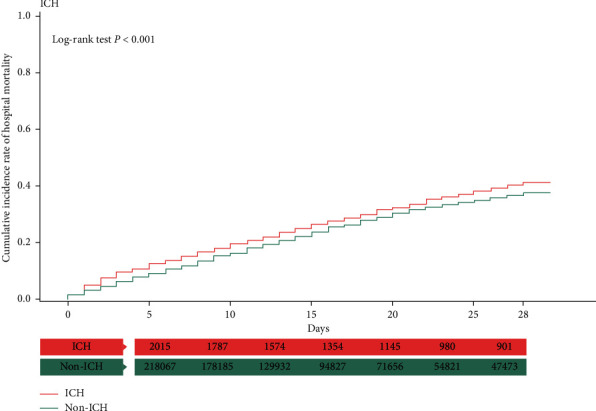
Kaplan–Meier analysis of cumulative hospital mortality in sepsis patients with and without ICH. The differences were evaluated by the log-rank test.

**Figure 3:**
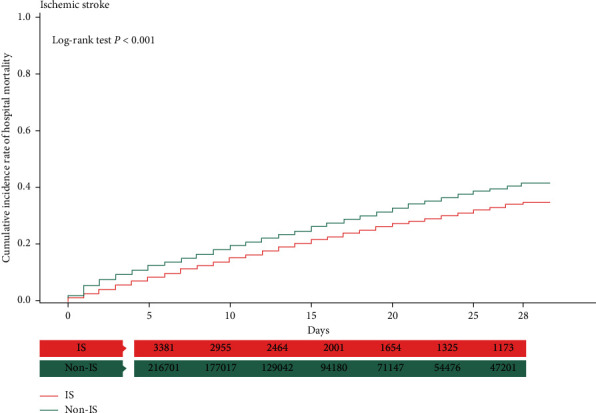
Kaplan–Meier analysis of cumulative hospital mortality in sepsis patients with and without ischemic stroke. The differences were evaluated by the log-rank test.

**Figure 4:**
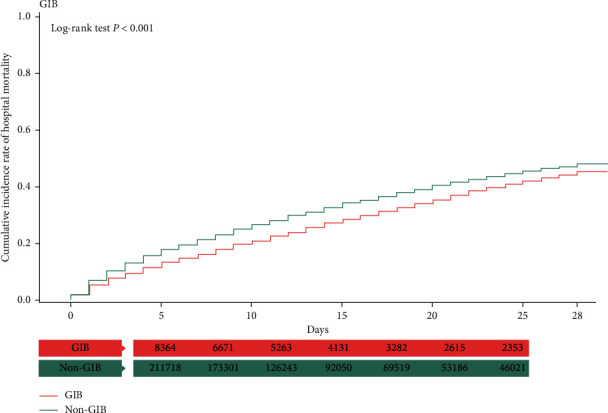
Kaplan–Meier analysis of cumulative hospital mortality in sepsis patients with and without GIB. The differences were evaluated by the log-rank test.

In the Kaplan–Meier analysis, the patients with sepsis complicated with AMI and ICH had a higher cumulative incidence of hospital mortality than those without AMI or ICH (log-rank test, *P* < 0.001) (Figures 1 and 2). However, the opposite phenomenon was observed in patients with ischemic stroke and GIB (log-rank test, *P* < 0.001) (Figures 3 and 4).

### 3.4. Joint Effect Analysis of GIB and MACE on Hospital Mortality, ICU Admission, and MV

The results of joint effect analysis shown in Table 3 summarize the sepsis outcomes if two or more complications occurred at the same time. The patients may have needed contrasting treatment during the sepsis course. For example, GIB needs hemostasis and cessation of antiplatelet drugs, and ischemic stroke needs antiplatelet drugs. GIB plus any MACE complicated the sepsis course by increasing ICU admissions and receiving MV. The combination did not affect total hospital mortality. Similar results were observed for other combinations such as AMI plus ischemic stroke. No specific combination of thrombotic complications such as ischemic stroke and AMI or hemorrhagic complications, such as GIB and ICH, significantly increased the risk of hospital mortality. However, the occurrence of more than one complication changed the clinical course, for example, by increasing the risk of ICU admission and MV. The combination of three or four complications was omitted because there were very few cases.

## 4. Discussion

To the best of our knowledge, this is the first and largest cohort study to comprehensively describe the individual and combined impact of GIB and MACE complications of sepsis in patients with a primary diagnosis of sepsis at the time of admission. Analysis of nationwide claims data in a cohort of sepsis patients found that GIB and MACE were associated with significantly increased risks of ICU admission and receiving MV for critical care and treatment of respiratory failure. Except for AMI and ICH, the complications did not affect mortality. In conclusion, GIB and MACE may not have a serious effect on hospital mortality as serious as was previously thought, and their occurrence will undoubtedly increase the complexity of the sepsis and hospital course. The use of preventive medications such as antiplatelet drugs, anticoagulants, statins, and PPIs should be monitored and balanced throughout the sepsis course.

### 4.1. Database Validation of the NHIRD

In clinical practice, this is a real-world condition that is encountered every day in the care of sepsis patients. Joint effect analysis provides a useful reference for physicians to predict the probable patient outcomes of sepsis complicated with multiple complications. In this nationwide database, GIB was the most frequent complication, occurred in 3.80% of the patients and followed by AMI in 1.59%, ischemic stroke in 1.54%, and ICH in 0.92%. The accuracy and reproducibility of this study is supported by the comparison with the hospital database (2006–2013) of Taichung Veterans General Hospital, a 1520-bed tertiary referral medical center in central Taiwan. The occurrence rate of GIB and MACEs was similar to that found in this study. In the hospital database, GIB occurred in 5.73% of the sepsis patients, followed by AMI (2.42%), ischemic stroke (1.54%), and ICH (1.22%).

### 4.2. AMI during Sepsis

An analysis of a national database in the USA by Smilowitz et al. in 2016 found that 118,183 of 2,602,854 sepsis inpatients (4.5%) had a concurrent diagnosis of AMI during hospitalization. The hospital mortality was higher in sepsis patients with concurrent AMI (aOR = 1.24), which is in line with our finding of an aOR of 1.11 (95% CI, 1.03–1.19) for sepsis complicated by AMI [10]. Both studies found that AMI not only complicated the sepsis course but was also associated with a significant increase in total hospital mortality. Smilowitz et al. reported that patients who were managed more invasively had much lower mortality than those managed conservatively.

### 4.3. Ischemic Stroke and ICH

Acute ischemic and hemorrhagic strokes are the fifth leading cause of death in the United States, and those who survive with subsequent long-term disabilities cause a heavy national socioeconomic burden [19]. Chronic hypertension, DM, atherosclerotic disease, and exposure to environmental toxins including air pollution have been identified as risk factors. A case-crossover study by Amelia et al. found that recent hospitalization for infection was associated with an increased risk of stroke, and that severe sepsis was associated with new-onset atrial fibrillation, which also increased stroke risk [20]. A population-based cohort study of inpatients in Denmark found that about 80% of the cardiovascular events in those admitted with bacteremia occurred within 6 months [21].

### 4.4. GIB during Sepsis

A database analysis of patients with septic shock in the USA by Siddiqui et al. reported that the incidence of GIB was 5.4% (6,571/119,684) in those hospitalized patients between 2003 and 2012. The occurrence of GIB was associated with a 9% increase in mortality from 45% to 54% [5]. In this study, the GIB occurred in 3.80% of the patients, and the occurrence of GIB increased mortality to 46% compared with 39% in uncomplicated sepsis. However, the adjusted OR did not find a significant association of GIB with an increased risk of mortality (aOR = 0.98, 95% CI, 0.93–1.03)). GIB is not an infrequent complication of sepsis patients, and it increases the complexity of care. Siddiqui et al. found that GIB increased the length of hospital stay from 15.76 to 20.56 days [5]. Consequently, effective GIB prophylaxis is important, and PPIs may be of use. A randomized controlled trial by Krag et al. comparing pantoprazole and placebo in critically ill patients at risk of GIB found that 90-day mortality. However, pantoprazole reduced the occurrence of clinically important GIB from 4.2% to 2.5% of the patients [22]. Although PPIs can prevent clinically important GIB, a recent meta-analysis by Alhazzani et al. concluded that routine use for stress ulcer prophylaxis may increase the risk of pneumonia, leaving their use during sepsis open to question [23]. A subsequent network meta-analysis by Wang et al. on the relationship of GIB and septic shock is in line with the results of this study that GIB increased the complexity of sepsis case management but did not influence hospital mortality [18].

### 4.5. Concurrence of GIB and MACEs

Joint effect analysis found that no combination of any two GIB and MACE complications had a significant effect on mortality as we previously thought, but any combination significantly increased the complexity of the sepsis course and the incidence of respiratory failure. If GIB or MACE complications were caused by sepsis and can be improved or resolved by supplemental therapy or prophylaxis, the complexity of the sepsis course would be reduced. However, hospital mortality would remain unchanged under the current best supportive care.

### 4.6. Limitations

This study had some limitations, but they did not detract from the correctness of our main study results. First, as a large epidemiologic study using an administrative database, it was inevitably lacking in laboratory data such as inflammatory markers and lactate levels. Composite outcomes, including total hospital mortality, ICU admission (a proxy of critical condition), and receiving MV (a proxy of respiratory failure), were used to estimate the impacts of GIB and MACE during sepsis because they were not affected by the laboratory data alone, but those outcomes reflected poor laboratory data. Second, in the administrative database, we could not distinguish whether PPIs were used for ulcer treatment or prophylaxis. However, prophylactic PPI use did not play a role in the sepsis course [24]. Third, different pathogens, such as bacteria, virus, and fungus, can influence the occurrence of associated complications. For example, systemic salmonella infection may cause abdominal aortic infected aneurysm [25]. However, in the NHIRD, information on the definite pathogen that caused sepsis was unavailable, except for some rare specific codings, for example, ICD-9-CM: 481, pneumococcal pneumonia; ICD-9-CM: 002, typhoid/paratyphoid fever; ICD-9-CM: 01, pulmonary tuberculosis [16].

## 5. Conclusion

GIB and MACE were associated with a significantly increased risk of ICU admission and MV but not with total hospital mortality, which was independently associated with AMI or ICH alone. Early prevention of GIB and MACEs can at least reduce the complexity of clinical course and even the hospital mortality.

## Figures and Tables

**Table 1:** Demographic characteristics and baseline comorbidities of sepsis patients.

Variables (*n* = 220,082)	Total		Hospital mortality				*P* value
			No		Yes		
	*n*	%	*n*	%	*n*	%	
Sex							<0.001
Female	95,982	43.61	62,472	46.47	33,510	39.13	
Male	12,4100	56.39	71,972	53.53	52,128	60.87	

Age group, years							<0.001
18–40 years	9078	4.12	6822	5.07	2256	2.63	
40–60 years	46,275	21.03	32,167	23.93	14,108	16.47	
60–80 years	97,665	44.38	60,655	45.12	37,010	43.22	
>80 years	67,064	30.47	34,800	25.88	32,264	37.67	
Mean (±SD)	70.15 (15.17)	68.32 (15.41)	73.00 (14.32)	<0.001			

Insurance premium (NT dollars)							<0.001
<20,000	138,504	62.93	79,070	58.81	59,434	69.4	
20,000–40,000	67,567	30.70	45,359	33.74	22,208	25.93	
40,000–60,000	10,328	4.69	7450	5.54	2878	3.36	
>60,000	3683	1.67	2565	1.91	1118	1.31	

Urbanization level							0.004
1 (highest)	53,004	24.08	32,181	23.94	20,823	24.32	
2	60,055	27.29	37,065	27.57	22,990	26.85	
3	36,139	16.42	22,030	16.39	14,109	16.48	
4	36,982	16.80	22,472	16.71	14,510	16.94	
5 (lowest)	33,900	15.40	20,695	15.39	13,205	15.42	

Baseline comorbidities							
HTN	15,0329	68.31	90,837	67.56	59,492	69.47	<0.001
DM	13,6875	62.19	83,590	62.17	53,285	62.22	0.825
CHF	58,264	26.47	32,176	23.93	26,088	30.46	<0.001
COPD	100,444	45.64	58,436	43.46	42,008	49.05	<0.001
CLD	67,061	30.47	40,663	30.25	26,398	30.83	0.004
CKD	82,200	37.35	46,355	34.48	35,845	41.86	<0.001
Cancer	69,432	31.55	35,366	26.31	34,066	39.78	<0.001

CCI score							<0.001
0	4748	2.16	3843	2.86	905	1.06	
1	10,415	4.73	8363	6.22	2052	2.4	
2	11,852	5.39	8744	6.5	3108	3.63	
3	13,081	5.94	9156	6.81	3925	4.58	
≥4	17,9986	81.78	104,338	77.61	75,648	88.33	

Drug use^¶^							
Aspirin	28,861	13.11	16,906	12.57	11,955	13.96	<0.001
Clopidogrel	17,667	8.03	9740	7.24	7927	9.26	<0.001
Warfarin	7325	3.33	4198	3.12	3127	3.65	<0.001
Metformin	48,257	21.93	33,803	25.14	14,454	16.88	<0.001
NSAIDs	151,508	68.84	95,432	70.98	56,076	65.48	<0.001
Statins	20,171	9.17	14,235	10.59	5936	6.93	<0.001
PPIs	91,831	41.73	45,818	34.08	46,013	53.73	<0.001
Steroids	118,048	53.64	59,845	44.51	58,203	67.96	<0.001
Immunosuppressants	1099	0.50	609	0.45	490	0.57	<0.001

Septic shock	111,754	50.78	43,310	32.21	68,444	79.92	<0.001
Endotracheal tube	73,098	33.21	27,647	20.56	45,451	53.07	<0.001
ICU admission	119,912	54.49	58,503	43.51	61,409	71.71	<0.001
Emergent hemodialysis	7600	3.45	2024	1.51	5576	6.51	<0.001
Hospital mortality rate	85,638	38.91					

GI bleeding							<0.001
No	21,1718	96.20	129,952	96.66	81,766	95.48	
Yes	8364	3.80	4492	3.34	3872	4.52	

Stroke							0.104
No	216,701	98.46	132,333	98.43	84,368	98.52	
Yes	3381	1.54	2111	1.57	1270	1.48	

ICH							<0.001
No	218,067	99.08	13,3346	99.18	84,721	98.93	
Yes	2015	0.92	1098	0.82	917	1.07	

AMI							<0.001
No	216,592	98.41	132,830	98.8	83,762	97.81	
Yes	3490	1.59	1614	1.2	1876	2.19	

Infection origins							
Central nervous	1382	0.63	870	0.65	512	0.60	0.153
Respiratory	87,748	39.87	47,674	35.46	40,074	46.79	<0.001
Cardiovascular	1614	0.73	1097	0.82	517	0.60	<0.001
Gastrointestinal/biliary	17,812	8.09	11,914	8.86	5898	6.89	<0.001
Genitourinary	66,518	30.22	50,467	37.54	16,051	18.74	<0.001
Soft tissue/musculoskeletal	10,960	4.98	8409	6.25	2551	2.98	<0.001
Device-related	3712	1.69	2729	2.03	983	1.15	<0.001
Others	17,651	8.02	12,923	9.61	4728	5.52	<0.001
Frequency of OPD visit^#^ (median, IQR)	18 (10–27)	17 (10–27)	18 (11–28)				
Frequency of ED visit^#^ (median, IQR)	18 (10–27)	17 (10–27)	18 (11–28)				

AMI, acute myocardial infarction; CCI score, Charlson comorbidity index score; CHF, congestive heart failure; CKD, chronic kidney disease; CLD, chronic liver disease; COPD, chronic obstructive pulmonary disease; ED, emergency department; GI, gastrointestinal; HTN, hypertension; ICH, intracranial hemorrhage; ICU, intensive care unit; IQR, interquartile range; NSAID, nonsteroidal anti-inflammatory drug; OPD, outpatient department; PPI, proton pump inhibitor; SD, standard deviation. ^¶^Use for more than 1 week within a one-year period prior to the index date. ^#^Within a one-year period prior to the index date.

**Table 2:** Impact of complications on the composite hospital outcomes.

Complications	*N*	Outcome = hospital mortality			Outcome = ICU admission			Outcome = mechanical ventilation		
		Event *n*	Event rate	Adjusted OR^¶^	Event *n*	Event rate	Adjusted OR^¶^	Event *n*	Event rate	Adjusted OR^¶^
				(95% CI)			(95% CI)			(95% CI)
GI bleeding										
No	211,718	81,766	0.39	1 (reference)	114,274	0.54	1 (reference)	69,314	0.33	1 (reference)
Yes	8364	3872	0.46	0.98 (0.93–1.03)	5638	0.67	1.30 (1.23–1.38)^*∗∗∗*^	3784	0.45	1.32 (1.26–1.40)^*∗∗∗*^

Stroke										
No	216,701	84,368	0.39	1 (reference)	117,396	0.54	1 (reference)	71,426	0.33	1 (reference)
Yes	3381	1270	0.38	0.84 (0.78–0.91)^*∗∗∗*^	2516	0.74	2.71 (2.47–2.97)^*∗∗∗*^	1672	0.49	2.07 (1.90–2.25)^*∗∗∗*^

ICH										
No	218,067	84,721	0.39	1 (reference)	118,096	0.54	1 (reference)	71,762	0.33	1 (reference)
Yes	2015	917	0.46	1.08 (0.98–1.19)	1816	0.90	8.02 (6.84–9.42)^*∗∗∗*^	1336	0.66	3.92 (3.52–4.40)^*∗∗∗*^

AMI										
No	216,592	83,762	0.39	1 (reference)	116,758	0.54	1 (reference)	70,941	0.33	1 (reference)
Yes	3490	1876	0.54	1.11 (1.03–1.19)^*∗∗∗*^	3154	0.90	4.78 (4.21–5.42)^*∗∗∗*^	2157	0.62	1.99(1.84–2.16)^*∗∗∗*^

Adjusted OR^¶^: adjusted for age, sex, insurance premium, urbanization level, and comorbidities in the logistic regression model. ^*∗∗∗*^*P* < 0.001.

**Table 3:** Joint effect analyses of association between “hospital mortality, ICU admission, and receiving mechanical ventilation” and “GI bleeding, stroke, ICH, and AMI.”

Complications				*N*	Outcome = hospital mortality			Outcome = ICU admission			Outcome = mechanical ventilation		
					Event	Event rate	Adjusted OR^¶^	Event	Event rate	Adjusted OR^¶^	Event	Event rate	Adjusted OR^¶^
GIB	Stroke	ICH	AMI				(95% CI)			(95% CI)			(95% CI)
No	No	No	No	203,522	77,972	0.38	1.00 (reference)	107,413	0.53	1.00 (reference)	64,600	0.32	1.00 (reference)
Yes	No	No	No	7965	3720	0.47	1.01 (0.96–1.07)	5285	0.66	1.30 (1.23–1.38)^*∗∗∗*^	3523	0.44	1.33 (1.26–1.40)^*∗∗∗*^
No	Yes	No	No	2990	1129	0.38	0.89 (0.82–0.97)^*∗∗*^	2162	0.72	2.62 (2.38–2.88)^*∗∗∗*^	1427	0.48	2.09 (1.91–2.28)^*∗∗∗*^
No	No	Yes	No	1748	821	0.47	1.17 (1.05–1.30)^*∗∗∗*^	1571	0.90	8.10 (6.84–9.60)^*∗∗∗*^	1158	0.66	4.11 (3.65–4.62)^*∗∗∗*^
No	No	No	Yes	3194	1738	0.54	1.15 (1.06–1.24)^*∗∗*^	2882	0.90	4.93 (4.33–5.62)^*∗∗∗*^	1956	0.61	2.03 (1.87–2.21)^*∗∗∗*^
Yes	Yes	No	No	139	44	0.32	0.50 (0.34–0.74)^*∗∗∗*^	118	0.85	3.79 (2.26–6.35)^*∗∗∗*^	81	0.58	2.38 (1.61–3.53)^*∗∗∗*^
Yes	No	Yes	No	106	33	0.31	0.53 (0.34–0.83)^*∗∗*^	96	0.91	8.12 (4.00–16.5)^*∗∗∗*^	74	0.70	4.84 (2.96–7.92)^*∗∗∗*^
Yes	No	No	Yes	134	67	0.50	0.82 (0.57–1.18)	119	0.89	3.07 (1.69–5.55)^*∗∗∗*^	92	0.69	2.61 (1.74–3.93)^*∗∗∗*^
No	Yes	Yes	No	115	41	0.36	0.70 (0.46–1.06)	106	0.92	12.41 (5.99–25.71)^*∗∗*^	72	0.63	3.42 (2.18–5.36)^*∗∗∗*^
No	Yes	No	Yes	112	46	0.41	0.68 (0.46–1.03)	106	0.95	12.28 (5.10–29.56)^*∗∗∗*^	77	0.69	3.48 (2.18–5.57)^*∗∗∗*^
No	No	Yes	Yes	31	16	0.52	1.05 (0.49–2.23)	29	0.94	7.37 (1.59–34.13)^*∗∗∗*^	22	0.71	3.29 (1.37–7.91)^*∗∗*^

Adjusted OR: adjusted for age, sex, insurance premium, urbanization level, and comorbidities in the logistic regression model. ^*∗∗*^*P* < 0.01; ^*∗∗∗*^*P* < 0.001. AMI, acute myocardial infarction; CI, confidence interval; GIB, gastrointestinal bleeding; ICH, intracranial hemorrhage; OR, odds ratio.

## Data Availability

The data that support the findings of this study are available from the LHDB but restrictions apply to the availability, which were used under license for the current study. They are not publicly available but are available from the corresponding author upon reasonable request.

## Abbreviations

AMI: Acute myocardial infarction
CI: Confidence interval
GIB: Gastrointestinal bleeding
ICD-9-CM: International classification of diseases, ninth revision, clinical modification
ICH: Intracranial hemorrhage
ICU: Intensive care unit
MACE: Major adverse cardiovascular event
NHIA: National Health Insurance Administration
NHIRD: National Health Insurance Research Database
OR: Odds ratio
PPI: Proton pump inhibitor.

## Additional Points

*Novelty Statement*. This study contributed at least several important novelties in clinical practice. (1) Here, we used the nationwide database of 15 years, from 1999 to 2013, including 220,082 patients, first admitted for sepsis to examine the impacts of two major common complications during sepsis, including gastrointestinal bleeding (GIB) and major adverse cardiovascular events (MACEs) on the hospital outcome. (2) We conducted the multivariate analysis to analyze the odds ratio of every complication on sepsis outcomes, including the hospital mortality, intensive care unit admission, and receiving mechanical ventilation. (3) Joint effect analysis of every complication of GIB and MACEs was conducted to clarify their interactions during sepsis.
